# Supplementary material for: Manipulation of Charge Delocalization in a Bulk Heterojunction Material Using a Mid-Infrared Push Pulse
Source: J Phys Chem C Nanomater Interfaces. 2023 Jul 6;127(28):13712–22. doi: 10.1021/acs.jpcc.3c02938 (PMC10364132; doi:10.1021/acs.jpcc.3c02938)
Supplement: Supplementary file 1 — jp3c02938_si_001.pdf [file jp3c02938_si_001.pdf]

Supporting Information for Publication

Manipulation of Charge Delocalization in a Bulk Heterojunction Material Using a Mid-Infrared Push Pulse

Angela Montanaro[1,2,•], Kyu Hyung Park[3,•], Francesca Fassioli[2,3,4], Francesca Giusti[1], Daniele Fausti[*,1,2], and Gregory D. Scholes[*,3]

[1] Department of Physics, University of Trieste, Via A. Valerio 2, 34127 Trieste, Italy; Elettra-Sincrotrone Trieste S.C.p.A. Strada Statale 14 - km 163.5 in AREA Science Park 34149 Basovizza, Trieste, Italy

[2] Department of Physics, University of Erlangen-Nürnberg, 91058 Erlangen, Germany

[3] Department of Chemistry, Princeton University, Princeton, New Jersey 08544, United States

[4] SISSA − Scuola Internazionale Superiore di Studi Avanzati, Trieste 34136, Italy

[•] *These authors contributed equally.*

AUTHOR INFORMATION

Corresponding Author: *gscholes@princeton.edu; *daniele.fausti@elettra.eu

Table of Contents

1. Experimental details

2. Computational details

3. Supplementary figures (Figure S1-S14)

4. References for supporting information

1. Experimental details

1.1. Chemicals and sample preparation

Poly(9,9-dioctylfluorene-alt-benzothiadiazole) (F8BT) ($M_n \leq$ 25,000 Da) and fullerene (C_{60}) (99.5%) were purchased from Sigma-Aldrich and used without further purification. F8BT and C_{60} were separately dissolved in chloroform (HPLC grade, ≥ 99%, contains amylene as stabilizer) by stirring 24 h, which were then mixed in equal weight ratio to prepare the blend film or diluted with the solvent to prepare the pristine F8BT film. Solutions were filtered out to remove any undissolved particles prior to spin coating. Fused quartz slides (2.54 × 2.54 cm^2) were cleaned by sonication in detergent solution (2 vol%, Hellmanex), deionized water (Milli-Q), acetone (HPLC grade, ≥ 99%), isopropanol (ACS reagent grade, ≥ 99.5%), dichloromethane (ACS reagent grade, ≥ 99.5%, contains amylene as stabilizer) for 20 min. each and, finally, by oxygen plasma treatment for 5 min.

1.2. Steady-state spectroscopy

Steady-state absorption and fluorescence spectra of the pristine F8BT and F8BT:C_{60} blend films, as well as F8BT solutions, were measured using Cary 100 UV-Visible spectrophotometer (Varian) and PTI QuantaMaster 400 (Horiba), respectively. Concentration of the F8BT solution samples were set to have absorbance < 0.5 to avoid the effect of self-absorption and self-quenching. Film samples tilted ~60° with respect to the incident excitation to direct emission to the detector in L-format, while avoiding collection of the reflected scatter.

1.3. Transient absorption spectroscopy

Pump-probe and pump-push-probe measurements were performed using two different experimental setups. The details of both setups are separately given in the following subsections.

1.3.1. Pump-probe spectroscopy

Description of the pump-probe setup is provided elsewhere.[1] The laser source is a Ti:Sapphire laser (Coherent Libra). Pump is tuned to 450 nm to resonate with the lowest-energy absorption band of F8BT for all samples discussed. The diameter of the pump and probe beam spots at the film or cuvette surface were 2 and 1 mm, respectively, which have been deliberately enlarged by placing the sample in front of the focal point. The pump intensity was varied systematically to test the dependence of excited-state dynamics on the pump fluence in the range of 0.4-3.2 μJ cm^{-2}. All measurements were performed at magic-angle configuration.

1.3.2. Pump-push-probe spectroscopy

Description of the pump-push-probe setup is provided elsewhere.[2] The light source employed is the Pharos laser by Light Conversion. Tunable mid-infrared (MIR) push pulse (70-250 meV) is generated by difference frequency generation in a GaSe crystal of two phase-locked near-infrared (NIR) pulses from a twin optical parametric amplifier (Orpheus TWIN, Light Conversion). Pump pulse is generated by second harmonic generation of a 20fs-long NIR output (900 nm) from non-collinear optical parametric amplifier (Orpheus-N, Light Conversion). A small portion (~1 μJ/pp) of the NIR output is used to generate the white-light (WL) probe by self-phase modulation in a sapphire crystal. Transmitted probe pulses are collected and dispersed by a blazed grating on a linear array of Si photodiodes (NMOS linear image sensor, Hamamatsu), whose pulse-by-pulse acquisition is synchronized with the laser repetition rate (5 kHz). Pump and push fluences were ~15 and ~500 μJ cm^{-2}, respectively. The implementation of a chopping system consisting of two optical blades synchronously running at known rotational frequencies (40 Hz for the pump and 20 Hz for the push) allows to separately acquire the dynamical response of the sample to: i) just the visible pump; ii) just the MIR push; iii) the joint response of both the pump and push. Direct

subtraction enables the calculation of the MIR-induced change in the pump-probe signal ($\Delta\Delta A = \Delta A^{push|ON} - \Delta A^{push|OFF}$). To prevent photodegradation, nitrogen was blown on the sample for the entire duration of the experiment.

1.4. Peak fitting procedure

Large inhomogeneous broadening of polymer systems makes Gaussian peak fitting a desirable method of capturing the time evolution of the peak position and intensity, but other functions, e.g., log-normal function, are also employed to fit asymmetric peaks. We used a combination of Gaussian (for ^{1}Ex, ^{3}Ex) and exponentially modified Gaussian functions[3] (for P2 and DP2) to analyze the pump-probe spectra in the visible and near infrared region. Peaks are assigned according to the time evolution characteristic to the identified excited-state species, also by carefully reviewing the literature, as detailed in the main text. Decomposed peaks are used to plot Figure 3, 4, and 6, which show one-to-one correspondence of the photogenerated species to their spectral signatures.

2. Computational details

All calculations are performed with a model oligomer consisting of four fluorene (F1) units and three benzothiadiazole (BT) units connected in an alternating fashion $(F1BT)_3$-F1 (hereafter referred to as trimer). Terminals of the trimer are capped with methyl groups and dioctyl groups in the fluorene unit of the original polymer are substituted with dimethyl groups to reduce the cost of computation. Ground-state geometry optimization, frequency calculation, vertical transition energy calculation, and natural transition orbital (NTO) simulation are performed at CAM-B3LYP/6-31G(d) level of theory using Gaussian(R) 16 software.[4] Optimized structures of neutral and radical cation trimers are shown in Figure S13 and S14.

3. Supplementary Figures

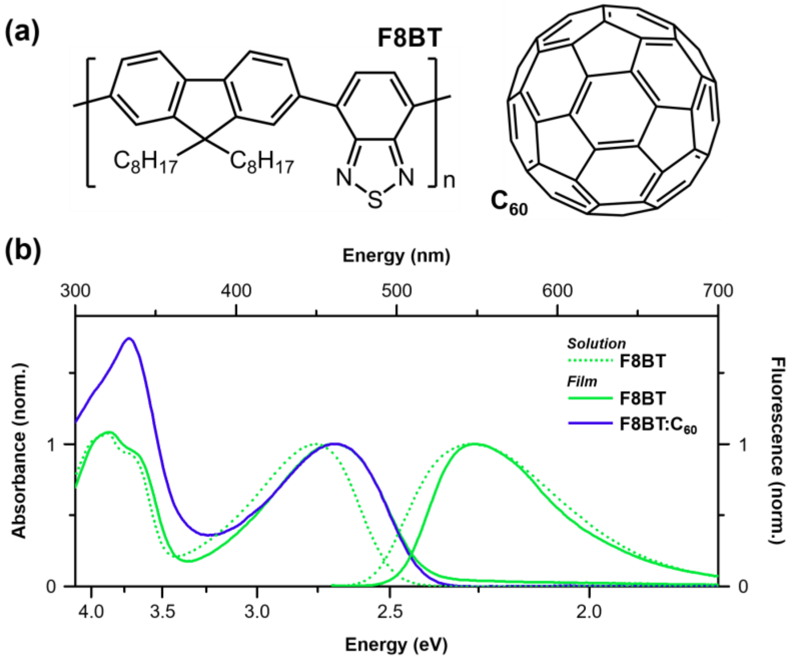

Figure S1. (a) Molecular structure of poly(9,9-dioctylfluorene-*alt*-benzothiadiazole) (F8BT) and fullerene (C_{60}). (b) Steady-state absorption and fluorescence spectra of F8BT in chloroform solution (green dotted lines), and F8BT pristine and F8BT:C_{60} blend films (green and blue solid lines, respectively).

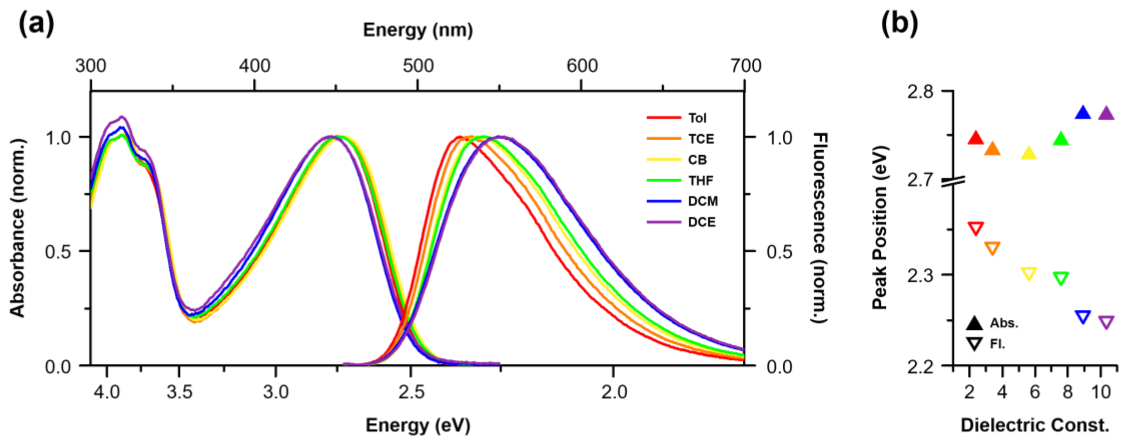

Figure S2. (a) Steady-state absorption and fluorescence spectra of F8BT in various solutions (Tol: toluene, TCE: trichloroethylene, CB: chlorobenzene, THF: tetrahydrofuran, DCM: dichloromethane, DCE: 1,2-dichloroethane). (b) Maxima of steady-state absorption (filled upward triangle) and fluorescence (empty downward triangle) bands as a function of dielectric constant of the solvent used.

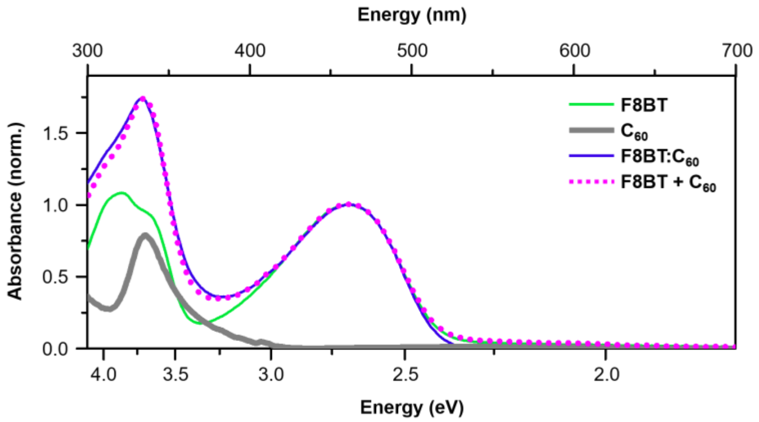

Figure S3. Steady-state absorption spectra of pristine F8BT (green solid line) and C_{60} blend (blue solid line) films. Simulated steady-state absorption spectrum of the blend film (pink dotted line) is obtained by adding the steady-state absorption spectra of the pristine film (green solid line) and C_{60} chloroform solution (grey solid line).

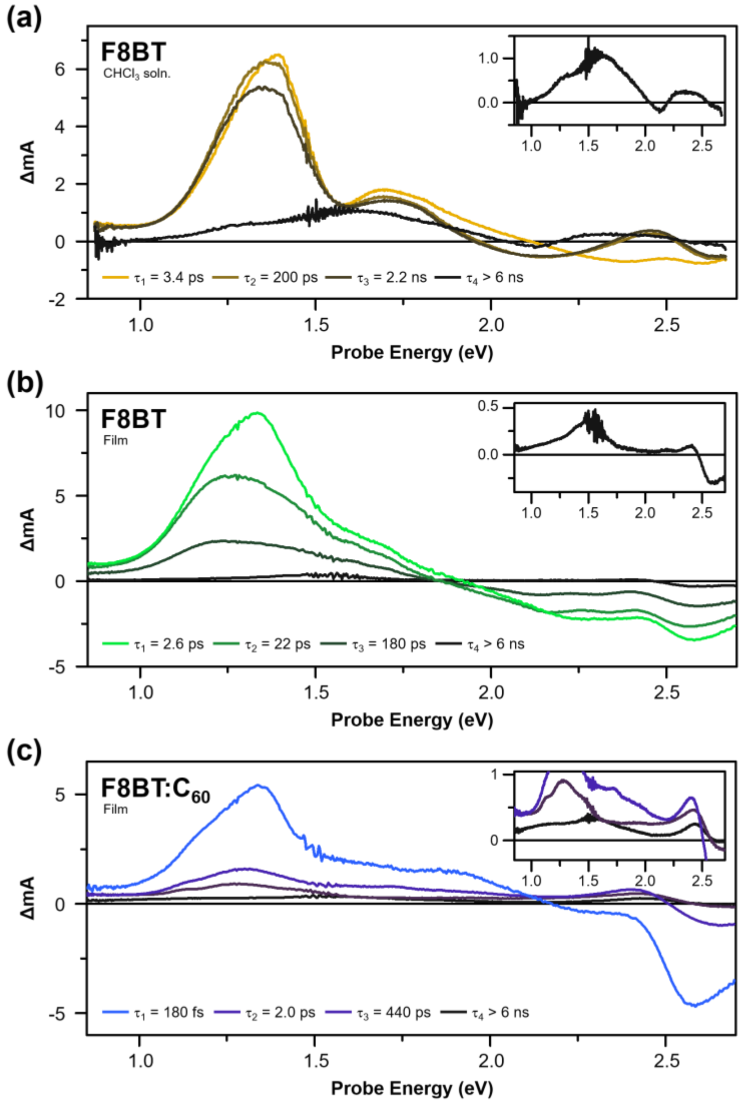

Figure S4. Evolution associated spectra of (a) F8BT in chloroform solution, (b) F8BT pristine film, and (c) F8BT:C_{60} blend film. Insets are scaled to show the spectra with small ΔA signals.

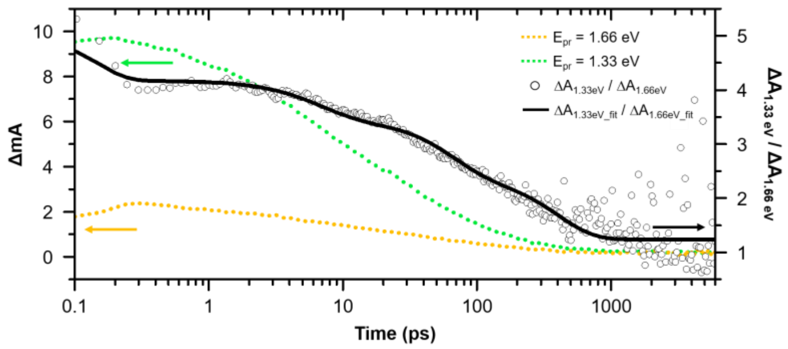

Figure S5. Decay traces of F8BT pristine film at probe energy 1.66 and 1.33 eV (yellow and green dotted line, respectively). These decay traces are analyzed with 4-exponential functions and the ratio of fitted curves (black solid line) are superimposed to the same plot from the raw data (○).

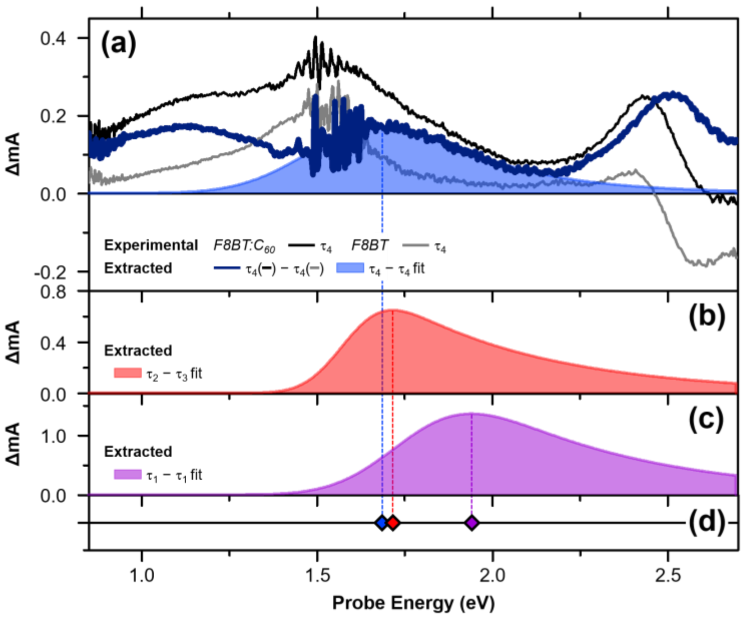

Figure S6. Excited-state absorption spectra corresponding to photogenerated $F8BT^{\bullet+}$ polaron in F8BT:C_{60} blend film associated with representative time constants, (a) τ_4, (b) τ_2, and (c) τ_1. The peak position (d) shifts significantly in going from τ_1 (◆; 1.94 eV) to τ_2 (◆; 1.72 eV), but only marginally from τ_2 (◆; 1.72 eV) to τ_4 (◆; 1.68 eV).

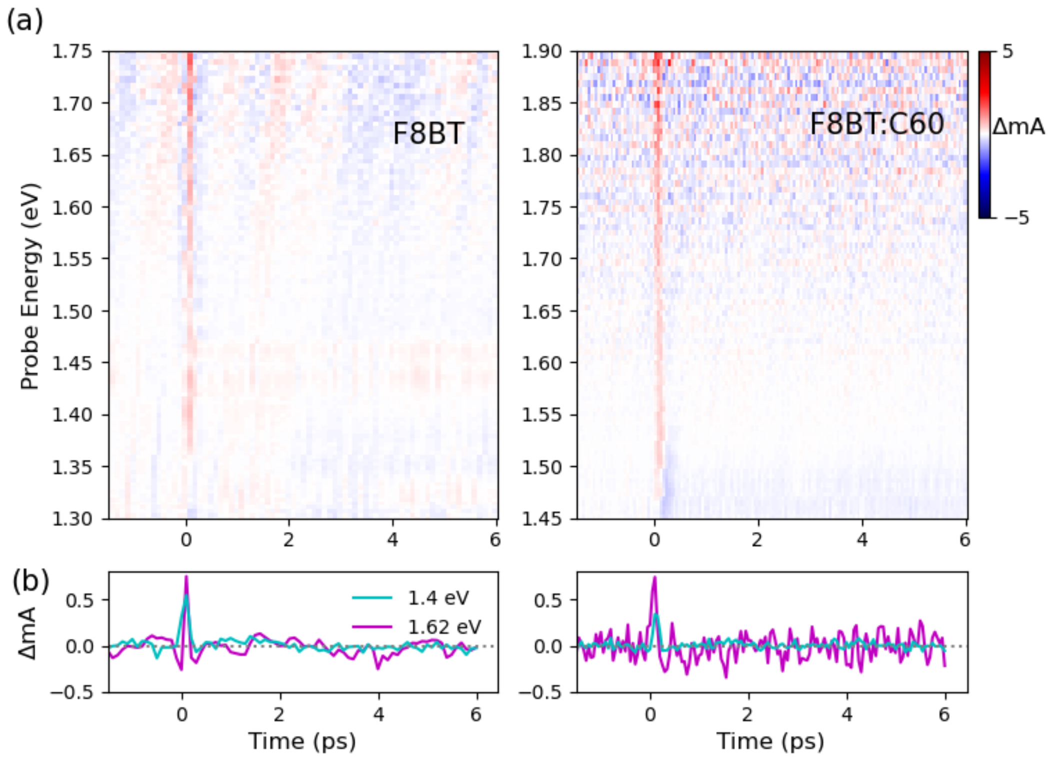

Figure S7. Push-probe maps as a function of push-probe delay (horizontal axis) and probe energy (vertical axis) on F8BT pristine (left panel) and F8BT:C_{60} blend film (right panel). The photon energy of the push pulse is 210 meV and its fluence 450 μJ cm^{-2}. (b) Time traces of the maps in (a) for two selected probe energies indicated in the legend.

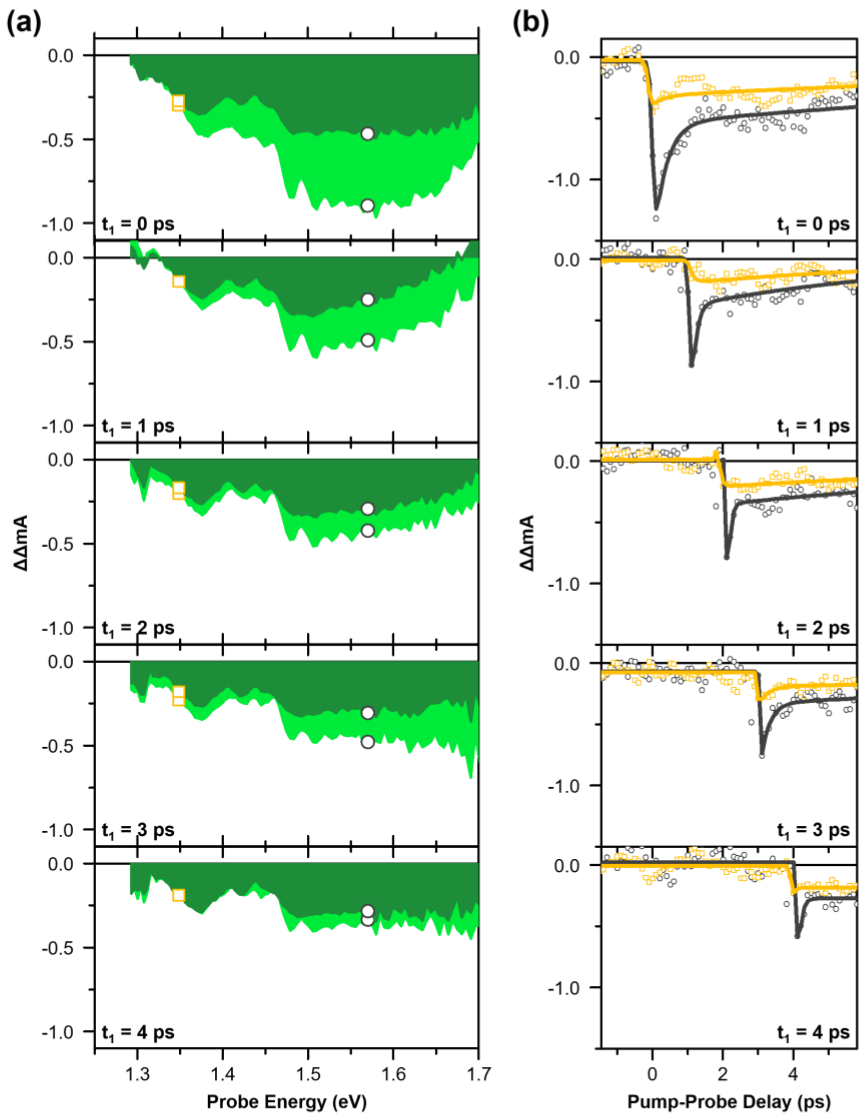

Figure S8. ΔΔ*A* (a) spectra and (b) time traces of F8BT pristine film as a function of pump-push time delay t_1. ΔΔ*A* spectra in light green shade (■) and dark green shade (■) are obtained by integrating push-probe time delay of 0.1−0.8 and 0.8−5.8 ps, respectively. The time traces are selected at representative probe energies indicated by the symbols in the spectra (○, 1.57 eV; □, 1.35 eV).

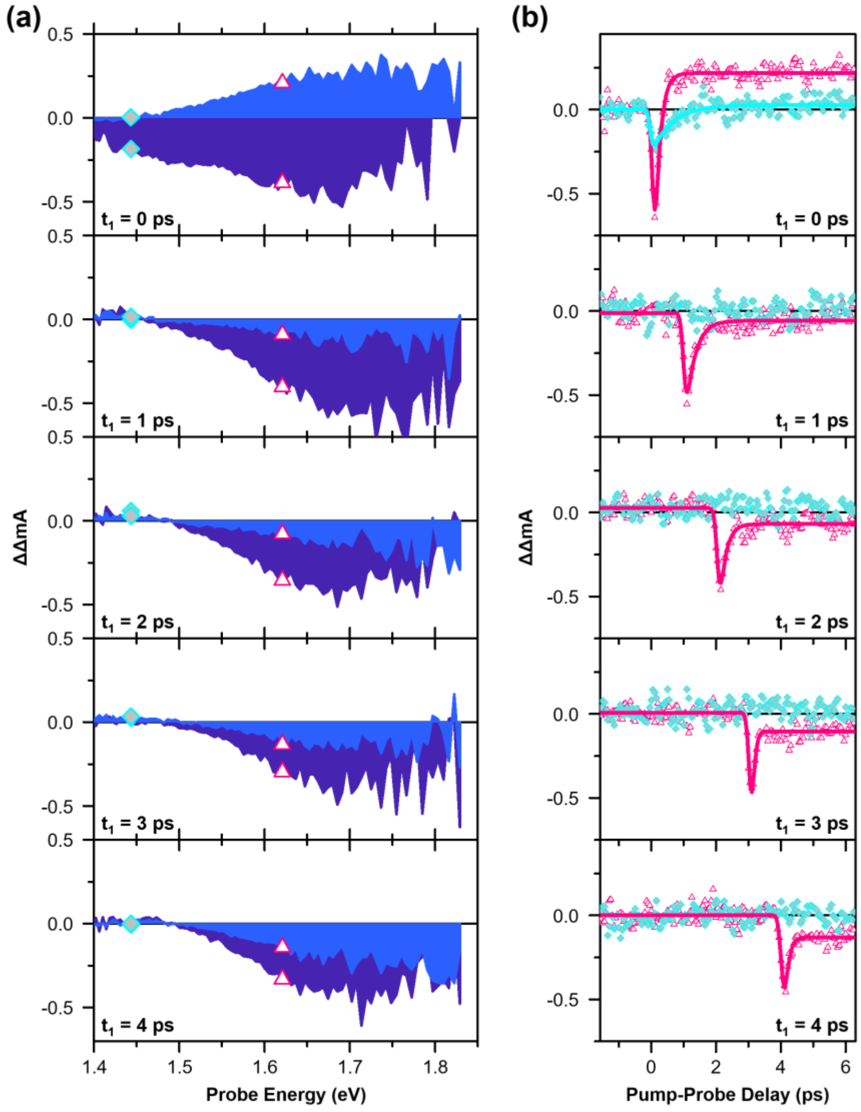

Figure S9. $\Delta\Delta A$ (a) spectra and (b) time traces of F8BT:C_{60} blend film as a function of pump-push time delay t_1. $\Delta\Delta A$ spectra in blue shade (■) and purple shade (■) are obtained by integrating push-probe time delay of 0.1−0.3 and 0.9−5.8 ps, respectively. The time traces are selected at representative probe energies indicated by the symbols in the spectra (△, 1.62 eV; ◇, 1.44 eV).

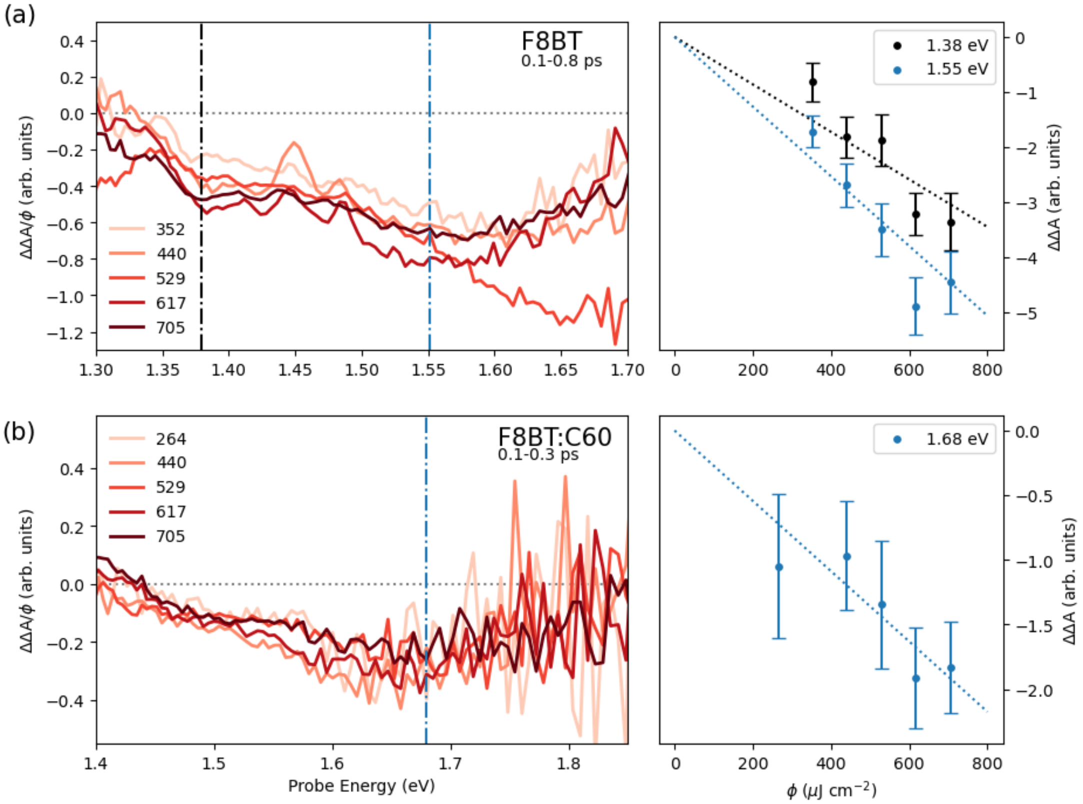

Figure S10. (a) Push-induced difference spectra (left panel) measured on the pristine F8BT film at different push fluences ϕ (indicated in the legend in units of μJ cm^{-2}). Each spectrum is integrated over the push-probe delay interval 0.1-0.8 ps and is divided by the corresponding fluence. In the right panel, the push-induced difference signal is plotted as a function of the push fluence for two selected probe energies (1.38 and 1.55 eV), marked in the left panel by the colored vertical lines. The dotted lines are linear fits to the two datasets. The error bars represent the standard deviations calculated for the selected probe energy over 5 repetitions. (b) As in (a), but for the F8BT:C_{60} blend film. The spectra are integrated over the push-probe interval 0.1-0.3 ps.

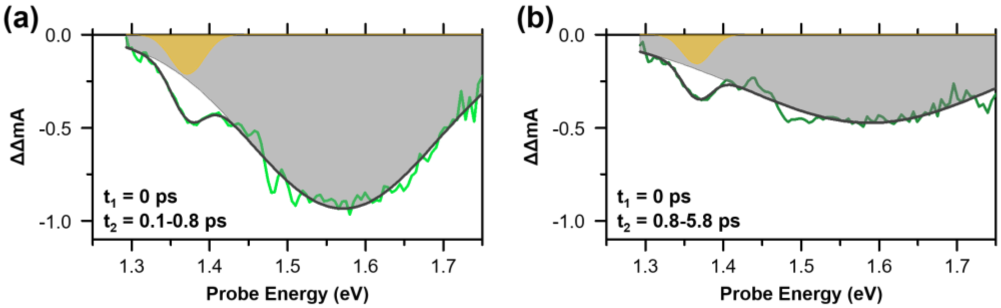

Figure S11. $\Delta\Delta A$ spectra of F8BT pristine film at pump-push delay t_1 = 0 ps and push-probe delay t_2 = (a) 0.1−0.8 ps (light green line) and (b) 0.8−5.8 ps (dark green line). Both spectra are fitted using two Gaussian peak functions (peak 1 and 2 in yellow and gray shades; sum of peak 1 and 2 in gray line). Center positions of both peaks (peak 1, 1.37 eV; peak 2, 1.58 eV) are invariant over push-probe delay t_2.

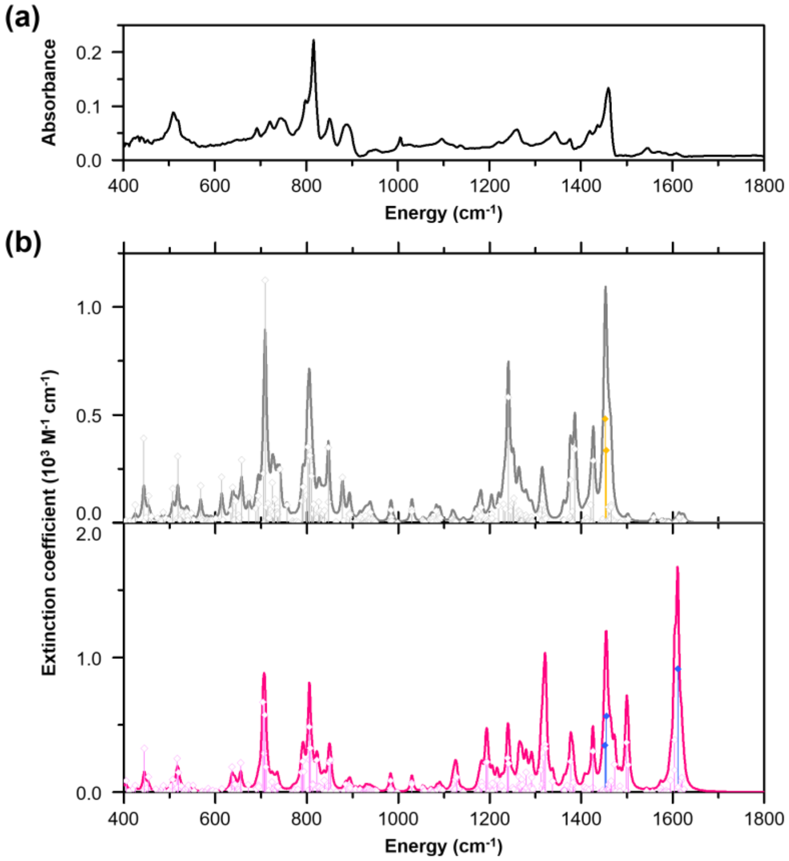

Figure S12. Steady-state infrared absorption spectrum of (a) neutral F8BT measured using ATR FT-IR spectroscopy. (b) Neutral (upper panel) and radical cation (lower panel) F8BT trimers (optimized structures shown in Figure S13 and S14) were used to calculate infrared extinction coefficients. The infrared spectra (solid line) are simulated by adding broadening of 8 cm^{-1} FWHM to all transitions (◇ with a vertical line).

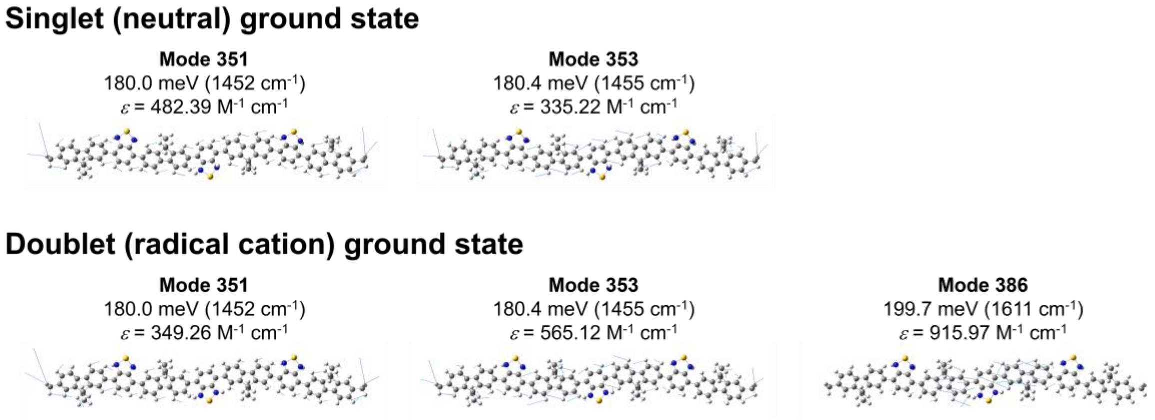

Figure S13. Vibrational modes that show strong push response (modes highlighted in Figure S12b). Thin blue arrows are displacement vectors of the constituent atoms.

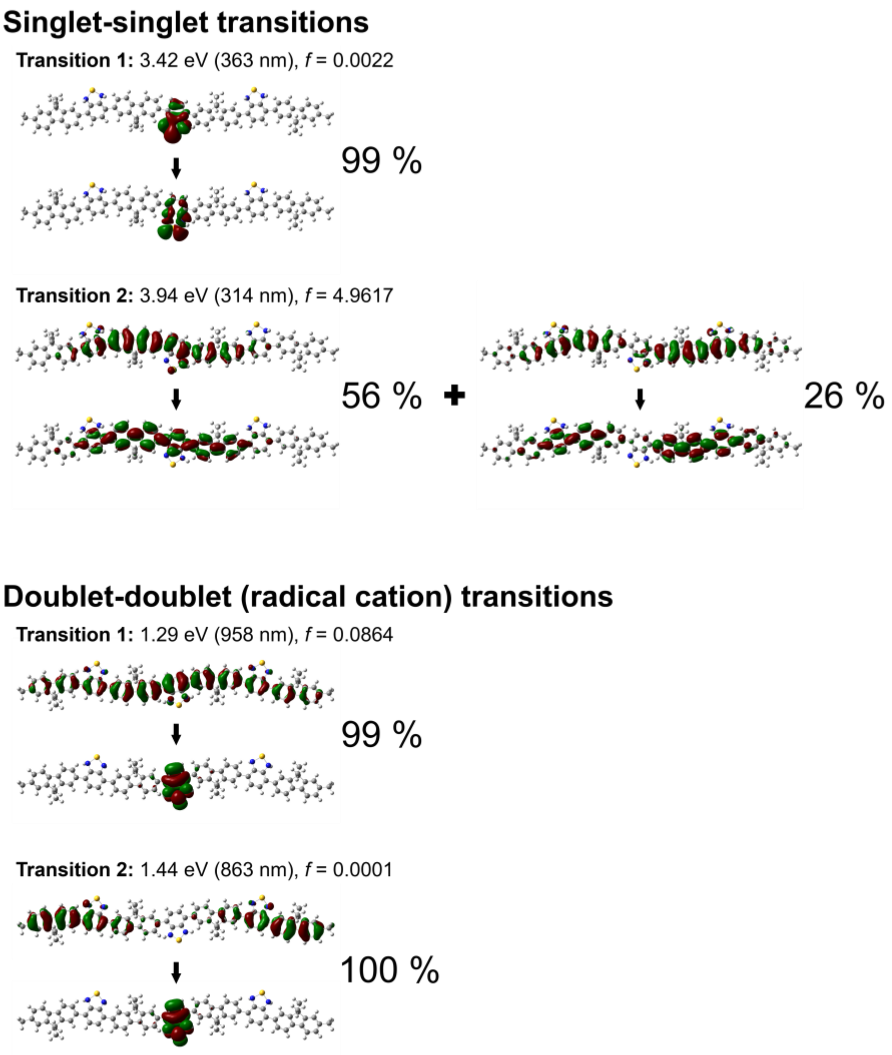

Figure S14. Representative natural transition orbitals (NTO) of the first two electronic transitions of neutral and radical cation F8BT trimers. Energy and oscillator strength of each transition are indicated above the NTO pair(s) that constitute the transition.

4. References for supporting information

(1) Pensack, R. D.; Ostroumov, E. E.; Tilley, A. J.; Mazza, S.; Grieco, C.; Thorley, K. J.; Asbury, J. B.; Seferos, D. S.; Anthony, J. E.; Scholes, G. D. Observation of Two Triplet-Pair Intermediates in Singlet Exciton Fission. *J. Phys. Chem. Lett.* **2016**, *7* (13), 2370–2375. https://doi.org/10.1021/acs.jpclett.6b00947.

(2) Montanaro, A.; Giusti, F.; Colja, M.; Brajnik, G.; Marciniak, A. M. A.; Sergo, R.; De Angelis, D.; Glerean, F.; Sparapassi, G.; Jarc, G.; et al. Visible Pump–Mid Infrared Pump–Broadband Probe: Development and Characterization of a Three-Pulse Setup for Single-Shot Ultrafast Spectroscopy at 50 KHz. *Rev. Sci. Instrum.* **2020**, *91* (7), 073106. https://doi.org/10.1063/5.0016362.

(3) Kalambet, Y.; Kozmin, Y.; Mikhailova, K.; Nagaev, I.; Tikhonov, P. Reconstruction of Chromatographic Peaks Using the Exponentially Modified Gaussian Function. *J. Chemom.* **2011**, *25* (7), 352–356. https://doi.org/10.1002/cem.1343.

(4) Frisch, M. J.; Trucks, G. W.; Schlegel, H. B.; Scuseria, G. E.; Robb, M. A.; Cheeseman, J. R.; Scalmani, G.; Barone, V.; Petersson, G. A.; Nakatsuji, H.; et al. Gaussian 16, Revision A.03. *Gaussian, Inc., Wallingford CT, 2016.*
